# Medical costs of treating myasthenia gravis in patients who need intravenous immunoglobulin (IVIg) – a register-based study

**DOI:** 10.1007/s00415-024-12768-5

**Published:** 2024-12-12

**Authors:** Christoffer Bugge, Ingrid Engebretsen, Ivar Sønbø Kristiansen, Erik Magnus Sæther, Ingrid Lindberg-Schager, Fredrik Arneberg, Nils Erik Gilhus

**Affiliations:** 1 Oslo Economics, Oslo, Norway; 2 Department of Health Management and Health Economics, Institute of Health and Society, University of Oslo, Forskningsveien 3A, 0373 Oslo, Norway; 3 Department of Public Health, Research Unit for General Practice, University of Southern Denmark, Campusvej 55, 5230 Odense M, Denmark; 4 UCB, Stockholm, Sweden; 5 UCB, Oslo, Norway; 6 Department of Clinical Medicine, University of Bergen, Bergen, Norway; 7 Department of Neurology, Haukeland University Hospital, Bergen, Norway

**Keywords:** Myasthenia gravis, Intravenous immunoglobulin, Healthcare costs, Registry data, Cost of illness

## Abstract

**Background:**

Several innovative treatments are expected for myasthenia gravis (MG) in the coming years. Healthcare payers usually require cost-effectiveness analyses before reimbursement. We aimed to investigate resource utilization and direct medical costs for patients with MG treated with intravenous immunoglobulin (IVIg) to inform such analyses.

**Methods:**

We identified patients with MG in the Norwegian Patient Registry based on at least two hospital encounters with an MG diagnosis (ICD-10 G70.0) from 1 Jan 2010 to 31 Dec 2021. IVIg treatment was identified by medical procedure and Anatomical Therapeutic Chemical (ATC) codes (RPGM05 and J06BA02). Using Diagnosis-Related Group (DRG) cost weights, we estimated direct medical costs for each year following the first MG diagnosis.

**Results:**

Over the study period, 1083 patients were diagnosed with MG in Norway, of whom 155 (14.3%) were treated with IVIg. No significant differences in age or sex were observed between IVIg and non-IVIg patients. Compared with non-IVIg patients, IVIg-patients had 2.3 times higher direct medical costs during the first year after MG diagnosis (EUR 35,714 vs. EUR 15,457) and 3.1 times higher costs during the second year (EUR 19,119 vs. EUR 6256). In the fifth year after diagnosis, IVIg-patients still had higher costs and resource utilization than non-IVIg patients (EUR 9953 vs. EUR 5634).

**Conclusion:**

IVIg treatment represents an important marker for high direct medical costs among patients with MG. The costs continue to be high during the first five years after MG diagnosis.

**Supplementary Information:**

The online version contains supplementary material available at 10.1007/s00415-024-12768-5.

## Introduction

Myasthenia gravis (MG) is a rare autoimmune disorder in which antibodies block or destroy acetylcholine receptors, lipoprotein-related protein 4 or MuSK antigens in the postsynaptic membrane at the neuromuscular junction [1]. Patients experience muscle weakness (weakness in the neck, shoulders and arms is common), fatigue, and problems with double vision, ptosis, swallowing and speech. Acetylcholinesterase inhibitors are sufficient to manage the mildest presentations of MG, but generalized MG usually requires long-term treatment with corticosteroids and immunosuppressants. Thymectomy is recommended for several MG subpopulations, for example for patients diagnosed with anti-acetylcholine receptor antibody-positive MG or patients with thymoma [2]. However, some individuals will experience myasthenic crises, which are characterized by respiratory muscle weakness, and require respiratory support and intensive care [1]. Intravenous immunoglobulin (IVIg) is usually used as short-term treatment for such crises or severe exacerbations [3], and is also sometimes used to induce a remission or to optimize the clinical condition before surgery or other medical challenges. IVIg is a biologic agent obtained through fractioning blood from healthy donors [4].

In a preceding study using nationwide data from Norway, we documented high costs associated with MG, not only for patients and the health care sector but also for society in general [5]. The annual mean costs in 2020 amounted to EUR 24,743 per patient, of which 15% were direct medical costs (in- and out-patient care and pharmaceuticals), 35% were costs related to productivity loss, and 51% stemmed from the value of lost life years and reduced health-related quality of life [5]. The direct medical costs were unevenly distributed among the patients. Therefore, mean cost per patient is not the optimal metric to illustrate the true economic burden of MG.

Differences in health care needs, resulting in an uneven distribution of medical costs among patients with MG, can be attributed to differences in patient and disease characteristics, such as the severity of muscle weakness, comorbidities, and age. Knowledge about factors contributing to cost variation in MG and the characteristics of patients with high treatment costs is limited. Studies from outside Europe indicate that IVIg treatment [6], hospitalizations [7, 8], and myasthenic crises [7] represent drivers of direct medical costs for patients with MG [3]. However, the applicability of these findings to European countries is limited because of differences in healthcare systems.

In the coming years, multiple innovative treatments for MG are expected to become available. Prior to reimbursement, healthcare payers will be likely to request cost-effectiveness analyses to evaluate value for money and to ensure an optimal allocation of limited healthcare resources. Furthermore, reimbursement is often restricted to specific disease subgroups where the treatment has been shown to be cost-effective. Consequently, public agencies and funding institutions need robust data on epidemiology, resource utilization, and direct medical costs to evaluate the cost-effectiveness of new treatments. Accurate estimates of direct medical costs for different MG subpopulations are, therefore, essential for improving the quality of care and ensure optimal resource allocation.

By utilizing nationwide patient-level registry data from Norway, we aimed to describe patient characteristics and estimate direct medical costs for the sub-cohort of patients with MG who received IVIg treatment compared with the MG population never treated with IVIg. This would determine whether IVIg treatment can be used as a marker for MG patients with especially high direct medical costs.

## Methods

### Study design

We conducted a non-interventional cohort study of patients diagnosed with MG, based on the use of mandatory, national registry data covering the entire Norwegian MG population.

### Data sources

We obtained nationwide patient-level data for all patients with at least one MG-related hospital encounter (recorded with ICD-10 G70.0) from 1 Jan 2008 to 31 Dec 2021 from the Norwegian Patient Registry (NPR). The NPR captures data on all treatment episodes in publicly funded hospitals (in- and outpatient care, including in-hospital drug use), thereby capturing the entire MG population in Norway. We obtained information on patient and treatment characteristics. Patient characteristics included sex, age, and date of death where applicable. Treatment characteristics included date of treatment, treatment level (inpatient or outpatient), length of stay for inpatient care, diagnosis-related groups (DRG) and their cost weights, main and contributory diagnosis (ICD-10 codes) at discharge from hospital, hospital drug treatment, and procedure codes. Population data were obtained from Statistics Norway [9].

### Identification of incident patients with MG and subgroups

Patients with MG were defined as individuals with at least two hospital encounters registered with an MG diagnosis (ICD-10 G70.0) from 2010 through 2021 [5]. Patients were followed from the time of the first MG diagnosis (index date) until the end of follow-up (death or end of study period). Patients without any MG-related hospital encounters during a two-year period, from 1 Jan 2008 to 31 Dec 2009, were considered incident patients with MG from 1 Jan 2010 to 31 Dec 2021. We excluded prevalent patients (those with an MG diagnosis in 2008 or 2009) and presented data only for the incident patients.

**Fig. 1:**
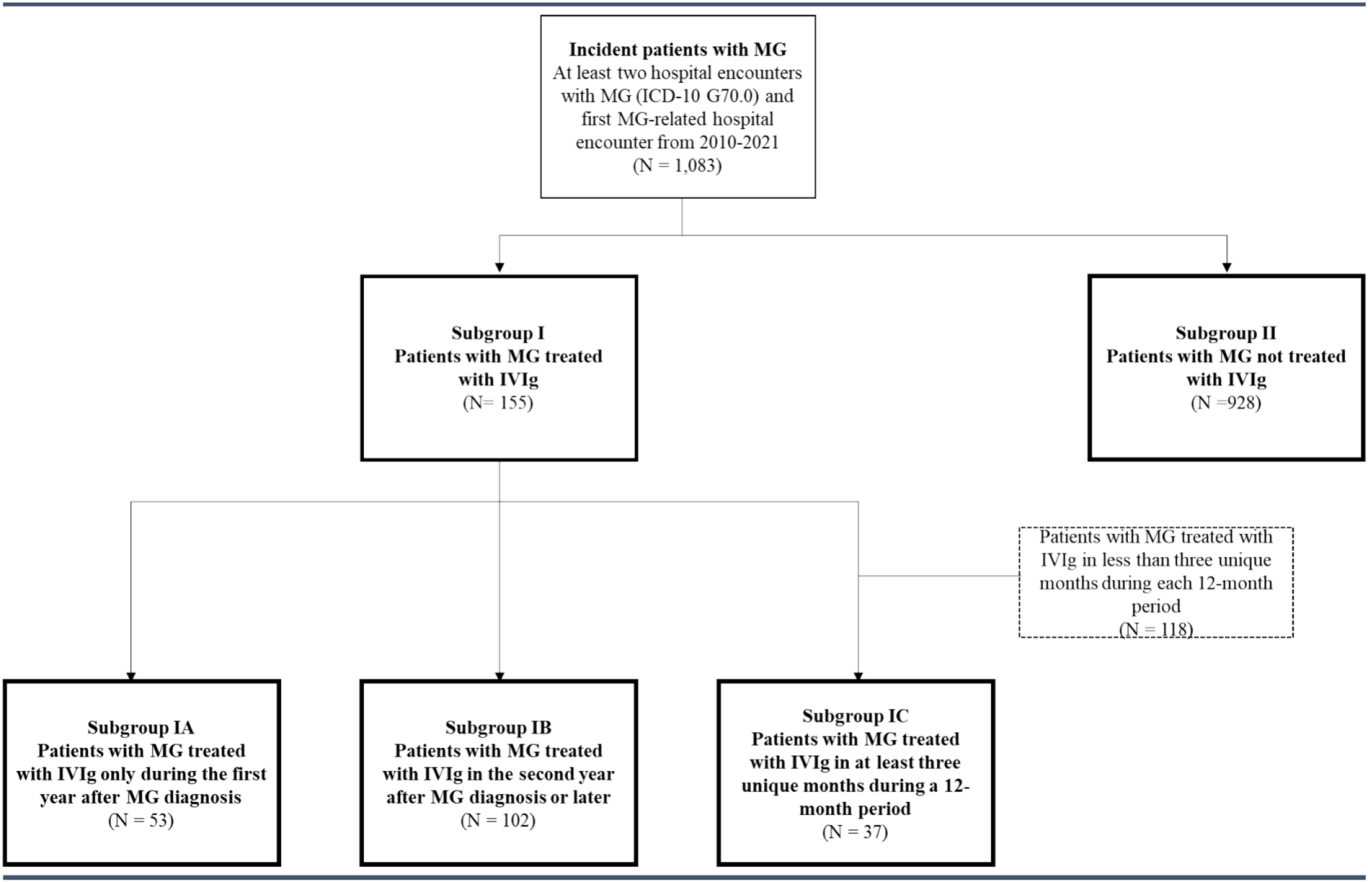
Study flow chart. *MG* myasthenia gravis, *IVIg* intravenous immunoglobulin

The incident patients with MG were divided into five subgroups (Fig. 1).

#### Subgroup I) IVIg-patients

Patients with at least one hospital encounter and Anatomical Therapeutic Chemical (ATC) code J06BA02 (immunoglobulin) or at least one hospital encounter with procedure code NCMP-code RPGM05 (infusion of gammaglobulin).

#### Subgroup IA) patients treated with IVIg only during the first year after MG diagnosis

Patients who were treated with IVIg during the first year following MG diagnosis and who did not receive IVIg in any following years.

#### Subgroup IB) patients treated with IVIg in the second year after MG diagnosis or later

Patients who undergo thymectomy may receive IVIg treatment prior to the surgery, which usually occurs during the first year after diagnosis. Furthermore, IVIg can be given during the first year to rapidly induce symptom improvement. Subgroup IB patients were those who were treated with IVIg during the second year following MG diagnosis or later (i.e. after the first year). The subgroup also included patients who received IVIg in the first year.

#### Subgroup IC) patients treated with IVIg as maintenance treatment

Some patients with MG receive IVIg as maintenance treatment. Reported intervals between IVIg cycles vary considerably, from 3 weeks up to several months [10–13]. To identify potential patients who receive IVIg as maintenance treatment, Subgroup IC was defined to include incident patients with at least one hospital encounter where IVIg was administered in at least three unique months during a 12-month period.

#### Subgroup II) non-IVIg patients

Patients with MG who had no recorded IVIg treatment during follow-up.

### Patient and characteristics

We describe patient characteristics in terms of annual MG incidence, sex, age, and the presence of thymoma, for the overall MG cohort and each subgroup. Thymoma was defined as at least one hospital encounter with the diagnostic code for thymoma (ICD-10 C37), either before or after first MG diagnosis.

### Study endpoints

We counted the total and mean number of hospital inpatient and outpatient encounters (i.e. treatment episodes). We calculated the direct medical costs (cost of inpatient and outpatient care and hospital administered drugs) related to treatment measured in monetary terms. We differentiated between costs specifically associated with MG (MG-related costs) and costs related to treatment of other medical conditions (non-MG-related costs). MG-related costs were identified as encounters with the diagnostic code for MG (ICD-10 G70.0) recorded as the primary or contributing diagnosis. The costs of non-MG-related encounters were identified as all encounters where MG was not listed as the primary or contributing diagnosis. Resource utilization and costs were analyzed across the entire follow-up period and, additionally, for each one-year interval following the initial MG diagnosis, for up to five years.

To estimate the direct medical costs, we applied DRG cost weights, following the same methodology as previously used by Bugge et al. [14]. All costs were adjusted for inflation to reflect 2021 EUR.

### Statistical analyses

We performed descriptive analyses only. Patient characteristics were presented in absolute numbers and as a proportion of patients (%). We present hospital encounters and resource utilization as mean number of encounters and mean (standard deviation [SD]) costs per patient. Analyses were conducted using R version 4.1.2 (2021).

### Ethics

Approval to use data from NPR were obtained from the National Research Ethics Committees (REK) (2021/102).

## Results

### Patient characteristics

We identified 1,083 incident patients with MG who had at least two MG-related hospital encounters from 2010 through 2021. Among these, 14.3% (*N* = 155) were treated with IVIg (IVIg patients), 4.9% (*N* = 53) were treated with IVIg in the first year only, and 9.4% (*N* = 102) were treated with IVIg in the second year or later (of which 31 patients (30.4%) received IVIg during the first year) (Table 1). 3.4% (*N* = 37) of the patients received IVIg in at least three unique months during a 12-month period (defined as maintenance treatment). In 2021, 18 patients with MG were treated with IVIg in at least three unique months (IVIg maintenance treatment). The male-to-female ratio was near to 1 in nearly all subgroups. The age distribution was similar among IVIg and non-IVIg patients.

**Table 1:** Patient and treatment characteristics for patients with MG according to IVIg treatment status, 2010–2021

	All MG	All IVIg patients	IVIg first year only	IVIg second or later years	IVIg maintenance	Non-IVIg patients
Number of patients	1,083	155	53	102	37	928
Mean annual incidence (per million per year)	17.5	2.5	0.9	1.6	0.6	15.0
Proportion female (%)	50.0	50.3	50.9	50.0	51.4	49.9
Median follow up (years from diagnosis to death or end of follow-up)	5.0	4.9	4.2	5.8	5.0	5.0
Age at first MG diagnosis, *N* (%)						
< 30 years	124 (11.4)	22 (14.2)	9 (17.0)	13 (12.7)	7 (18.9)	102 (11.0)
30–39 years	92 (8.5)	11 (7.1)	–	10 (9.8)	5 (13.5)	81 (8.7)
40–49 years	124 (11.4)	18 (11.6)	–	14 (13.7)	–	106 (11.4)
50–59 years	139 (12.8)	14 (9.0)	–	10 (9.8)	10 (27.0)	125 (13.5)
60–69 years	235 (21.7)	41 (26.5)	14 (26.4)	27 (26.5)	6 (16.2)	194 (20.9)
70–79 years	238 (22.0)	33 (21.3)	11 (20.8)	22 (21.6)	–	205 (22.1)
80 + years	131 (12.1)	16 (10.3)	10 (18.9)	6 (5.9)	0 (0.0)	115 (12.4)
Thymoma, *N* (%)	40 (3.7)	6 (3.9)	–	5 (4.9)	–	34 (3.7)

Groups with less than five observations are not presented due to confidentiality considerations (–)

*IVIg* intravenous immunoglobulin

### Healthcare resource utilization and costs

From the first MG diagnosis (index date) until end-of follow-up, the patients had a mean of 4.4 inpatient hospital stays and 9.1 outpatient encounters (Table 2). The numbers were similar among males and females (Supplementary Table 5). Patients younger than 50 years at the time of MG diagnosis had more outpatient encounters than those aged ≥ 50 years. Older patients (≥ 50 years of age) received more inpatient care due to longer hospital stays and thus accumulated higher costs per patient (Supplementary Table 5).

**Table 2:** Direct medical costs in hospital and resource utilization, MG-related episodes, 2010–2021

	All MG (*N* = 1083)	All IVIg patients (*N* = 155)	IVIg first year only (*N* = 53)	IVIg second or later years (*N* = 102)	IVIg maintenance (*N* = 37)	Non-IVIg patients (*N* = 928)
Inpatient care						
Number of hospital stays	4787	1530	396	1134	433	3257
Mean [SD] number of hospital stays per patient	4.4 [6.9]	9.9 [11.4]	7.5 [8.3]	11.1 [12.6]	11.7 [11.5]	3.5 [5.3]
Mean [SD] length of stay (days)	6.8 [10.1]	6.7 [10.1]	7.9 [11.9]	6.3 [9.3]	6.5 [10.3]	6.8 [10.2]
Outpatient care						
Number of outpatient encounters	9873	3188	616	2572	1244	6685
Mean [SD] number of outpatient encounters per patient	9.1 [12.3]	20.6 [24.9]	11.6 [10.0]	25.2 [28.8]	33.6 [40.1]	7.2 [6.8]
Cost of hospital encounters						
Total costs (million EUR), MG-related hospital contacts	41.7	14.8	3.8	11.0	4.1	26.9
Total costs (million EUR), MG-related hospital contacts excl. IVIG episodes	36.6	9.7	2.7	6.9	2.3	26.9
Mean [SD] cost (EUR) per patient, MG-related hospital contacts	38,457 [72,954]	95,364 [120,628]	71,934 [108,380]	107,538 [125,306]	111,148 [135,563]	28,952 [56,240]
Mean [SD] cost (EUR) per patient, MG-related hospital contacts excl. IVIG episodes	33,936 [63,808]	64,974 [93,080]	55,461 [94,575]	69,779 [92,426]	68,059 [78,811]	28,952 [56,240]
Mean [SD] cost (EUR) per patient, MG-related hospital contacts for patients with at least one MG-related inpatient stay	49,990 [80,117]	99,790 [121,685]	71,935 [108,381]	99,790 [121,685]	128,330 [138,153]	39,119 [62,818]

*MG* myasthenia gravis, *IVIg* intravenous immunoglobulin

For IVIg patients, the mean number of inpatient stays during the whole follow-up was 9.9, and they had a mean of 20.6 outpatient encounters (Table 2). For IVIg-patients treated after the first year, the mean number of inpatient stays and outpatient encounters were even higher, at 11.1 and 25.2, respectively. For patients who received IVIg as maintenance treatment, the mean number of inpatient stays was 11.7, while it was 33.6 for outpatient encounters. The mean number of IVIg-hospital encounters was 8.8 for all IVIg patients, 2.1 for those treated first year only, 12.3 for those treated with IVIg second year or later, and 28.7 for those on maintenance treatment. Among all IVIg patients, 47.0% had more than two encounters with IVIg, while 27.7% had more than five encounters with IVIg (Supplementary Table 6).

Among all patients with MG, the mean MG-related hospital cost was EUR 38,457 per patient for the whole follow-up period (Table 2). For IVIg patients, it was EUR 95,364, while it was EUR 107,538 for patients treated with IVIg after the first year. IVIg patients on maintenance treatment had mean MG-related costs of EUR 111,148. For patients not treated with IVIg treatment, the mean per-patient cost of MG-related encounters was EUR 28,952.

The mean number of non-MG-related inpatient hospital stays was 4.3 for IVIg patients, 5.1 for patients treated with IVIg after the first year, and 3.1 for patients not treated with IVIg (Supplementary Table 1). The mean number of non-MG outpatient encounters during the same period was 28.1 for all IVIg-patients, 33.6 for patients treated with IVIg after the first year, and 19.7 for patients not treated with IVIg.

**Fig. 2:**
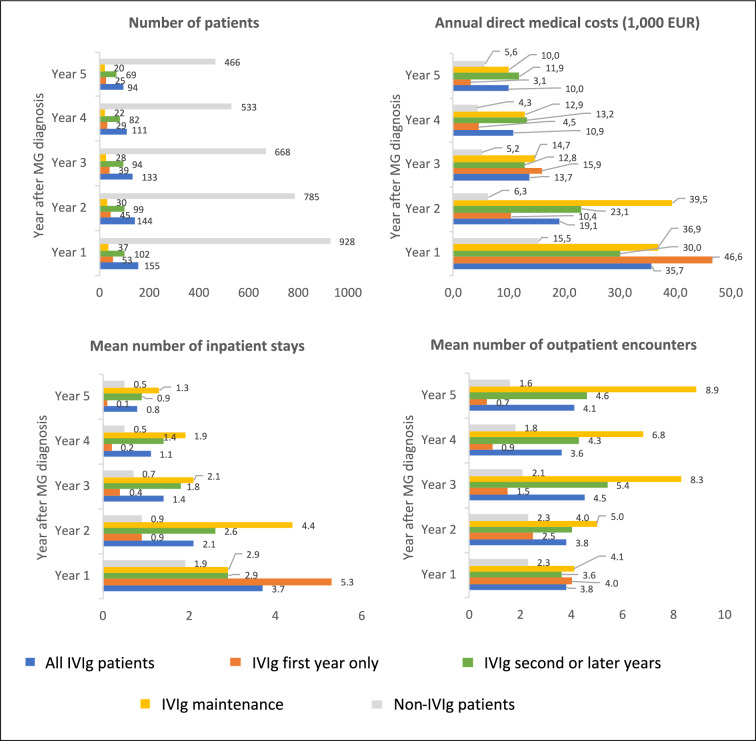
Resource utilization and costs of MG-related hospital contacts per patient, by IVIg treatment status and year after first MG diagnosis (MG-related hospital encounters and costs only), 2010–2021. *IVIg* Intravenous immunoglobulin

IVIg patients had a mean of 3.7 MG-related inpatient hospital stays and 3.8 MG-related outpatient encounters during the first year following MG diagnosis (Fig. 2). Non-IVIg patients had 1.9 inpatient stays and 2.3 outpatient encounters during that year. The annual per-patient cost of hospital encounters during the first year for patients treated with IVIg was EUR 35,714, while it was EUR 15,457 for patients not treated with IVIg. For the second and third years after MG diagnosis, patients treated with IVIg had an annual per-patient cost of MG-related hospital encounters of EUR 19,119 and EUR 13,747, respectively, compared with EUR 6256 and EUR 5170 for the non-IVIg group. For the fifth year following MG diagnosis, the costs were EUR 9953 for patients treated with IVIg, and EUR 5634 for patients not treated with IVIg. Patients treated with IVIg only in the first year had the highest costs and resource utilization in the first year, but lower in all subsequent years.

The non-MG-related cost of in-patient and out-patient hospital care for IVIg patients was EUR 8364 per patient during the first year following the MG diagnosis. For those treated with IVIg after the first year it was EUR 9012 and for non-IVIg patients it was EUR 6820 (Supplementary Table 3). During the third year after an MG diagnosis, the non-MG related costs were EUR 5960 for patients treated with IVIg, EUR 6913 for patients treated with IVIg after the first year, and EUR 6204 for patients not treated with IVIg.

## Discussion

In this long-term registry study of incident patients with MG in Norway, we identified a large variation in healthcare utilization and costs across patient subgroups. Patients undergoing IVIg treatment had higher resource utilization and costs during all five years after the MG diagnosis compared with non-IVIg patients.

A key strength of this study is the use of nationwide data covering the entire Norwegian population following patients for up to 12 years. The presence of a public health care system with almost no patient co-payments and universal access, combined with a long history of collecting data in mandatory public registries, ensures data collection for all individuals residing in the country. This eliminates selection bias and lack of representativeness and allows us to accurately identify patients with MG and IVIg treatment and their costs.

Still, our study has limitations. First, the diagnostic codes used in the NPR have not been specifically validated for MG. However, the validity of diagnostic codes has been established through comparisons with other disease-specific quality registries [15]. While a first MG diagnostic code can be recorded while physicians wait for confirmation or rejection of the diagnosis, the final diagnostic sensitivity and specificity are likely to be high because we required at least two encounters with an MG diagnosis (our criteria prioritized specificity over sensitivity [5]). Second, our study only considered resource utilization and healthcare costs related to hospital treatment. Primary care resource utilization, patient-administered drug use, and other societal costs were not included due to lack of data. As a result, overall healthcare utilization and costs are likely to be higher. Lastly, we were not able to formally evaluate the severity of the MG disease in our study population.

We show that IVIg treatment is an important marker for high healthcare utilization and direct medical costs associated with MG. Previous studies have reported IVIg in MG as a marker of high costs in North America [6], with mean per-patient hospital costs for IVIg patients in the USA of USD 78,814 in 2011, and in Canada of Canadian Dollars (CAD) 8310 in 2014 [16, 17]. Another US-based study from 2010 reported a median per-patient hospital charge of USD 21,124 for patients with MG receiving IVIg [18]. A study from the US encompassing 7194 patients during 2010–2019 found that the clinical burden in terms of severe exacerbations and crises was highest in the first year after diagnosis [19]. In this study, 33% of patients were hospitalized during the first year, with declining proportions down to 16% during the fifth year [19]. The increased MG-related costs are most pronounced during the first years after diagnosis. Our results give a more detailed assessment as the previous studies lacked information about the duration of the follow-up period for which costs were assessed [17], did not report patient numbers [16], and gave no information on the year of currency valuation [16, 18]. Further, the Norwegian health care system differs substantially in organization and funding from those of North America, leading to differences in treatment routines and treatment costs. Our results clearly show the importance of IVIg as a driver of MG-related resource utilization and medical costs for a public health care system.

The direct medical costs and the related resource utilization were highest during the first year after MG diagnosis for all patients, regardless of IVIg use, in line with the results from a recent study [20]. Patients with MG are sometimes hospitalized for muscle weakness of unknown cause before their MG diagnosis. In patients younger than 50 years, thymectomy is frequently undertaken during the initial year after diagnosis [21]. Understanding how resource utilization and costs vary across patient populations and with time after diagnosis will be important for decision-makers when trying to estimate the cost-effectiveness of new treatment options for MG. Such evaluations should be based on the relevant MG characteristics and cost estimates as reported in this study.

Despite similar age and sex distributions in the two subgroups, IVIg patients had considerably higher resource utilization and costs than the non-IVIg group. Across the entire follow-up period, both MG-related inpatient stays and outpatient encounters were 2.8-fold higher for IVIg patients, compared with the non-IVIg group. The difference in the corresponding direct medical costs was even larger: IVIg patients had 3.3-fold higher direct medical costs during follow-up. IVIg treatment is invasive, may have side effects, and is costly. Even when we excluded costs related to treatment episodes where IVIg were administered (i.e. all costs related to in- or outpatient hospital treatment), IVIg patients had 2.2-fold higher direct medical costs during follow-up compared to the non-IVIg group. IVIg is mostly given as treatment for MG crisis and severe exacerbations. This explains why this treatment is a relevant marker for MG severity. IVIg is also sometimes given before thymectomy and other surgical procedures, whereas it is rarely given as long-term treatment in Norway [22]. In this study, 3.4% of patients receive IVIg maintenance treatment and 9.4% received IVIg in the second year after diagnosis or later. Plasma exchange represents an alternative treatment for acute exacerbations and severe MG. In Norway, plasma exchange is rarely used compared to IVIg and is therefore probably a less useful marker to define high-cost MG subgroups. Immunoglobulin can be given subcutaneously instead of intravenously, which is typically for longer-term use. According to data from the National Prescription Registry, only 28 patients with MG (< 2%) received subcutaneous immunoglobulin in Norway between 2008 and 2021.

Our results illustrate that IVIg treatment is an important marker of direct medical costs associated with MG. Changes in these costs should be accounted for when evaluating new therapeutic interventions. IVIg patients are overrepresented among those with the highest direct medical costs, reflecting that these patients probably have a more severe disease. Innovative treatments for MG will likely become available in the coming years. Accurate information on the current standard of care is needed for the optimal assessment of new therapies. Good information ensures valid comparisons between old and new therapies. Our study presents new and important information regarding cost drivers and estimates to be used in models evaluating the cost-effectiveness of new treatments.

## Conclusion

IVIg treatment is a marker of high medical costs in patients with MG. The expected and real changes in such costs should be accounted for when evaluating new interventions.

## Supplementary Information

Below is the link to the electronic supplementary material.

Supplementary file1 (DOCX 77 KB)

## Data Availability

The data that support this study are not publicly available due to ethical restriction.
