# Supplementary material for: Medical costs of treating myasthenia gravis in patients who need intravenous immunoglobulin (IVIg) – a register-based study
Source: J Neurol. 2024 Dec 12;272(1):15. doi: 10.1007/s00415-024-12768-5 (PMC11638270; doi:10.1007/s00415-024-12768-5)
Supplement: Supplementary file 1 — Supplementary file1 (DOCX 77 KB) [file 415_2024_12768_MOESM1_ESM.docx]

# Supplementary materials

Supplementary Table 1: Direct medical costs and resource utilization estimated from hospital encounters for non-MG indications, 2010-2021

|  | | All MG (N=1,083) | All IVIg  patients (N=155) | IVIg first year only  (N=53) | IVIg second or later years (N=102) | IVIg maintenance (N=37) | Non-IVIg patients (N=928) |
| --- | --- | --- | --- | --- | --- | --- | --- |
|  | Inpatient care | | | | | | |
| Number of hospital stays | | 3,565 | 659 | 919 | 518 | 621 | 2,906 |
| Mean [SD] number of hospital stays per patient | | 3.3 [8.0] | 4.3 [15.3] | 2.7 [3.6] | 5.1 [18.7] | 16.8† [35.5] | 3.1 [6.0] |
| Mean [SD] length of stay (days) | | 4.1 [6.4] | 4.0 [7.3] | 3.3 [3.9] | 4.2 [8.0] | 4.0 [7.8] | 4.1 [6.2] |
|  | Outpatient care | | | | | | |
| Number of outpatient encounters | | 22,649 | 4,347 | 141 | 3,428 | 3131 | 18,302 |
| Mean [SD] number of outpatient encounters per patient | | 20.9 [33.0] | 28.1 [40.3] | 17.3 [23.3] | 33.6 [45.9] | 84.6 [90.0] | 19.7 [31.5] |
|  | Cost of hospital encounters | | | | | | |
| Total costs (million EUR), all episodes from first MG diagnosis | | 30.4 | 5.9 | 1.1 | 4.8 | 3.3 | 24.5 |
| Mean [SD] cost per patient (EUR), all episodes from first MG diagnosis | | 29,284 [70,469] | 38,868 [133,376] | 21,114 [26,168] | 47,833 [162,103] | 88,780 [258,342] | 27,638 [52,560] |

†Mean is influenced by one patient with very high resource use. MG = Myasthenia Gravis, IVIg = Intravenous immunoglobulin

Supplementary Table 2: Direct medical cost and resource utilization, estimated for hospital encounters for all indications (MG and non-MG), 2010-2021

|  | | All MG (N=1,083) | All IVIg  patients (N=155) | IVIg first year only  (N=53) | IVIg second or later years (N=102) | IVIg maintenance (N=37) | Non-IVIg patients (N=928) |
| --- | --- | --- | --- | --- | --- | --- | --- |
|  | Inpatient care | | | | | | |
| Number of hospital stays | | 8,352 | 2,189 | 537 | 1,652 | 1,054 | 6,163 |
| Mean [SD] number of hospital stays per patient | | 7.7 [11.0] | 14.1 [19.4] | 10.1 [9.9] | 16.2 [22.7] | 28.5  [35.0] | 6.6 [8.4] |
| Mean [SD] length of stay (days) | | 5.6 [8.8] | 5.9 [9.4] | 6.7  [10.1] | 5.6 [8.9] | 5.0 [9.0] | 5.6  [8.6] |
|  | Outpatient care | | | | | | |
| Number of outpatient encounters | | 32,522 | 7,535 | 1,535 | 6,000 | 4,375 | 24,987 |
| Mean [SD] number of outpatient encounters per patient | | 30.0  [36.1] | 48.6  [48.9] | 29.0  [26.2] | 58.8  [54.6] | 118.2  [90.1] | 26.9 [32.6] |
|  | Cost of hospital encounters | | | | | | |
| Total costs (million EUR), all episodes from first MG diagnosis | | 72.0 | 20.7 | 4.9 | 15.8 | 7.4 | 51.3 |
| Mean [SD] cost per patient (EUR), all episodes from first MG diagnosis | | 66,497 [107,319] | 133,479  [185,190] | 92,252 [114,687] | 154,902 [210,153] | 199,928 [284,811] | 55,310 [82,886] |

MG = Myasthenia Gravis, IVIg = Intravenous immunoglobulin

Supplementary Table 3: Resource utilization and direct medical costs of non-MG indicated hospital encounters for patients with MG, by subgroup and year after first MG diagnosis

|  | Hospital encounters for non-MG indications | | | | | | | | | | | | | | | | | | | |
| --- | --- | --- | --- | --- | --- | --- | --- | --- | --- | --- | --- | --- | --- | --- | --- | --- | --- | --- | --- | --- |
|  | Number of patients | | | | | Mean number of inpatient stays | | | | | Mean number of outpatient encounters | | | | | Annual cost of hospital encounters per patient  (EUR) | | | | |
|  | All IVIg patients | IVIg first year only | IVIg second or later years | IVIg main-tenance | Non-IVIg patients | All IVIg patients | IVIg first year only | IVIg second or later years | IVIg main-tenance | Non-IVIg patients | All IVIg patients | IVIg first year only | IVIg second or later years | IVIg main-tenance | Non-IVIg patients | All IVIg patients | IVIg first year only | IVIg second or later years | IVIg main-tenance | Non-IVIg patients |
| Year 1 | 155 | 53 | 102 | 37 | 928 | 0.8 | 0.9 | 0.7 | 1.2 | 0.7 | 5.2 | 4.6 | 5.5 | 8.6 | 3.5 | 8,364 | 7,135 | 9,012 | 19,179 | 6,820 |
| Year 2 | 144 | 45 | 99 | 30 | 785 | 0.7 | 0.6 | 0.7 | 1.7 | 0.6 | 6.7 | 5.0 | 7.1 | 13.4 | 3.8 | 7,251 | 5,185 | 8,271 | 16,005 | 5,935 |
| Year 3 | 133 | 39 | 94 | 28 | 668 | 0.6 | 0.1 | 0.7 | 1.6 | 0.5 | 4.0 | 1.8 | 4.6 | 7.8 | 4.2 | 5,960 | 2,954 | 6,913 | 12,192 | 6,204 |
| Year 4 | 111 | 29 | 82 | 22 | 533 | 0.7 | 0.3 | 0.8 | 2.4 | 0.6 | 3.9 | 1.3 | 4.5 | 10.5 | 3.8 | 8,174 | 5,674 | 8,916 | 18,449 | 6,760 |
| Year 5 | 94 | 25 | 69 | 20 | 466 | 1.0 | 0.2 | 1.1 | 3.4 | 0.6 | 4.1 | 1.5 | 4.4 | 9.5 | 3.5 | 10,490 | 5,088 | 12,759 | 29,195 | 6,307 |

MG = Myasthenia Gravis, IVIg = Intravenous immunoglobulin

Supplementary Table 4: Resource utilization and direct medical costs of all hospital encounters (MG and non-MG) for patients with MG, by subgroup and year after first MG diagnosis

|  | Hospital encounters for all indications | | | | | | | | | | | | | | | | | | | |
| --- | --- | --- | --- | --- | --- | --- | --- | --- | --- | --- | --- | --- | --- | --- | --- | --- | --- | --- | --- | --- |
|  | Number of patients | | | | | Mean number of inpatient stays | | | | | Mean number of outpatient encounters | | | | | Annual cost of hospital encounters per patient  (EUR) | | | | |
|  | All IVIg patients | IVIg first year only | IVIg second or later years | IVIg main-tenance | Non-IVIg patients | All IVIg patients | IVIg first year only | IVIg second or later years | IVIg main-tenance | Non-IVIg patients | All IVIg patients | IVIg first year only | IVIg second or later years | IVIg main-tenance | Non-IVIg patients | All IVIg patients | IVIg first year only | IVIg second or later years | IVIg main-tenance | Non-IVIg patients |
| Year 1 | 155 | 53 | 102 | 37 | 928 | 4.5 | 6.2 | 3.7 | 4.1 | 2.6 | 8.9 | 8.6 | 9.1 | 12.6† | 5.8 | 43,376 | 53,217 | 38,263 | 53,496 | 21,094 |
| Year 2 | 144 | 45 | 99 | 30 | 785 | 2.6 | 1.5 | 3.0 | 6.1 | 1.2 | 10.1 | 7.6 | 10.6 | 18.5 | 5.5 | 22,936 | 13,699 | 27,135 | 48,483 | 9,240 |
| Year 3 | 133 | 39 | 94 | 28 | 668 | 1.9 | 0.5 | 2.4 | 3.6 | 1.0 | 8.1 | 3.3 | 9.6 | 16.1 | 5.6 | 17,760 | 17,040 | 18,058 | 24,715 | 8,526 |
| Year 4 | 111 | 29 | 82 | 22 | 533 | 1.7 | 0.5 | 1.9 | 4.3 | 0.9 | 6.9 | 2.2 | 8.0 | 17.4 | 5.0 | 15,215 | 7,555 | 17,924 | 27,623 | 8,142 |
| Year 5 | 94 | 25 | 69 | 20 | 466 | 1.6 | 0.4 | 1.9 | 4.6 | 0.9 | 7.4 | 2.1 | 9.0 | 18.4 | 4.6 | 15,971 | 6,410 | 19,434 | 31,368 | 8,436 |

†Mean is influenced by one patient with very high resource use. MG = Myasthenia Gravis, IVIg = Intravenous immunoglobulin

Supplementary Table 5: Resource utilization and direct medical costs (EUR) of hospital care for MG, 2010-2021, by sex and age group at diagnosis

|  | Female <50 y. (N=225) | Female≥ 50 y. (N=316) | Male < 50 y. (N=115) | Male≥50 y. (N=427) |
| --- | --- | --- | --- | --- |
| Inpatient care | | | | |
| Number of hospital stays | 1,045 | 1,321 | 537 | 1,884 |
| Mean [SD] number of hospital stays per patient | 4.6 [7.4] | 4.2 [6.2] | 4.7 [10.3] | 4.4 [5.9] |
| Mean [SD] length of stay (days) | 4.8 [8.1] | 7.4 [11.3] | 5.0 [6.1] | 7.9 [10.9] |
| Outpatient care | | | | |
| Number of outpatient encounters | 2,781 | 2,168 | 1,203 | 3,721 |
| Mean [SD] number of outpatient encounters per patient | 12.4 [18.9] | 6.9  [8.9] | 10.5 [15.5] | 8.7 [7.9] |
| Cost of hospital encounters | | | | |
| Total cost (million EUR), MG-related hospital contacts | 6.7 | 13.0 | 4.0 | 17.9 |
| Mean [SD] cost per patient (EUR), MG-related hospital contacts | 29,818 [40,629] | 41,262 [80,661] | 34,853 [76,311] | 41,904 [78,868] |

Supplementary table 6: Number of hospital encounters where IVIg was administered, by subgroup, 2010-2021

|  | All IVIg  patients (N=155) | IVIg first year only  (N=53) | IVIg second or later years (N=102) | IVIg maintenance (N=37) |
| --- | --- | --- | --- | --- |
| Mean [SD] number of hospital encounters with IVIg | 8.8 [18.1] | 2.1 [1.2] | 12.3 [21.5] | 28.7 [29.3] |
| Median [IQR] number of hospital encounters with IVIg | 2 [4.5] | 2  [1] | 5 [11.3] | 22 [27] |
| Patients with more than two hospital encounters with IVIg, N (%) | 77 (47.0) | 12 (22.6) | 65 (63.7) | 37 (100) |
| Patients with more than five hospital encounters with IVIg, N (%) | 43 (27.7) | - | 42 (41.2) | 34 (91.9) |

Groups with Less than five observation are not presented due to confidentiality considerations (-). IVIg = Intravenous immunoglobulin
